# What Is Wrong With the Current Evaluative Bibliometrics?

**DOI:** 10.3389/frma.2021.824518

**Published:** 2022-01-21

**Authors:** Endel Põder

**Affiliations:** Institute of Psychology, University of Tartu, Tartu, Estonia

**Keywords:** bibliometric indicators, research evaluation, multi-authorship, fractionalized counting, individual researcher's performance, number of coauthors, research culture

## Abstract

Bibliometric data are relatively simple and describe objective processes of publishing articles and citing others. It seems quite straightforward to define reasonable measures of a researcher's productivity, research quality, or overall performance based on these data. Why do we still have no acceptable bibliometric measures of scientific performance? Instead, there are hundreds of indicators with nobody knowing how to use them. At the same time, an increasing number of researchers and some research fields have been excluded from the standard bibliometric analysis to avoid manifestly contradictive conclusions. I argue that the current biggest problem is the inadequate rule of credit allocation for multiple authored articles in mainstream bibliometrics. Clinging to this historical choice excludes any systematic and logically consistent bibliometrics-based evaluation of researchers, research groups, and institutions. During the last 50 years, several authors have called for a change. Apparently, there are no serious methodologically justified or evidence-based arguments in the favor of the present system. However, there are intractable social, psychological, and economical issues that make adoption of a logically sound counting system almost impossible.

## Introduction

During the past few decades, the quantitative measurement of scientific performance has started to play an important role. Counts of publications, citations, and h-index are frequently used to evaluate work of scientists. However, many scientists feel that these measurements don't capture important aspects of their work and may be heavily misleading sometimes. Also, experts in bibliometrics have no clear answers to apparently simple and practical questions. They like to emphasize that there is no one correct indicator and that it is better to use several (e.g., Bornmann and Marx, 2014). They also recommend taking into account different contextual factors not included in the indicators themselves (Panaretos and Malesios, 2009; Hicks et al., 2015). One may conclude that measurement of scientific performance is very complex and necessarily subjective.

On the other hand, bibliometric data are relatively simple and describe objective processes of publishing articles and citing others. It seems quite straightforward to define reasonable measures of a researcher's productivity, research quality, or overall performance based on these data. Of course, the simple measures may ignore possibly important details, but they should be logically consistent and understandable and not lead to obviously contradictive results.

I argue that the current biggest problem is the inadequate rule of credit allocation for multiple authored articles in mainstream bibliometrics. The basic bibliometric indicators were conceived when most scientific papers had a single author. In that condition, publication and citation counts might have worked well, and ranking of researchers based on productivity and impact could be simple and straightforward. Multiple authorship requires one more basic step—division of credits between co-authors.

## Credit Allocation With Multiple Authors—Elephant in the Room of Bibliometrics

According to common sense, when a group of individuals creates something, credit is divided among them. However, that does not apply to scientific papers. In mainstream bibliometrics, each of the multiple authors of an article claims full credit, as if he (or she) has completed the whole study alone. This odd practice necessarily leads to problems.

First, it is grossly unfair. To add five publications to his list of publications, a solo author has really to write five. A member of a group of five coauthors has in average to do 1/5 of this work, or about one article in total.

Second, this kind of evaluation creates a strong motivation to join bigger groups. Nowadays, every researcher knows that collaboration is a key to success. Publishing articles together with a colleague, you can easily double your numbers of publications and citations as compared to publishing individually, and organizing a group of 10 collaborators gives, in average, a 10 fold advantage to all of them. Given such a strong incentive, it is not surprising that research groups are growing fast.

Two recent articles on the credit allocation problems in bibliometrics have independently used the same metaphor of “the elephant in the room” in their title (Lozano, 2013; Waltman et al., 2016). Perhaps this reflects some feeling about the presence of a big and obvious problem that people still are trying not to see.

Really, the problem of multiple authors has a strange status in bibliometrics. The problem has been noticed, and a way to correct it was proposed several decades ago (Lindsey, 1980; Price, 1981). However, no practical measures have been taken since. Sometimes, the problem has been discussed in theoretical works but has been largely unknown to wider community. In this century, several independent researchers from different fields (Schreiber, 2008; Põder, 2010; Lozano, 2013; Vavryčuk, 2018) have rediscovered the problem and called to correct it. Still, neither the researchers of bibliometrics nor institutions providing bibliometric indicators have shown interest to either follow the proposal or present convincing arguments against it.

## Ordinary Researchers, Experts of Bibliometrics, and Publishers of Indicators

Like other people, researchers are interested in feedback on their work and comparison of achievements of himself and others. Bibliometric indicators offer a simple and amusing way for that. Moreover, universities and financing bodies are increasingly using bibliometrics to evaluate performance of researchers.

Ordinary researchers acquire their knowledge of bibliometrics from well-known publishers of scientific information—Web of Science (Clarivate), Google Scholar, or Scopus (Elsevier). All these organizations present only the traditional whole-count statistics as indicators related to an individual researcher. Unsuspecting users are led to believe that this is the best professional bibliometrics can offer. Still, the same databases could easily support counting of coauthors and calculation of weighted publication and citation scores that are free from inflation bias caused by multi-authorship. Up to now, only Harzing's Publish or Perish has options to calculate fractionalized publication and citation scores and unbiased versions of h-index.

Assumed experts of bibliometrics seem not to want to take any strong position and avoid directly addressing the problem. Several authors (e.g., Waltman, 2016; Sivertsen et al., 2019) have cited an idea from Moed (2005) that different indicators measure different aspects of performance: fractionalized indicators measure contribution, and full-count indicators measure participation. However, given that distinction, we may think further what we really want to measure. I believe that there is no question—from these two options, scientific contribution fits better the purpose of evaluative bibliometrics to provide metrics for unbiased measurement of productivity, or impact, of a researcher. Then, why don't proceed with this one?

In recent years, many studies have used fractionalized indicators to compare performance of universities or countries (e.g., Schneider, 2009; Aksnes et al., 2017), and researchers generally believe that these indicators give more reasonable and consistent results (Huang et al., 2011; Aksnes et al., 2012; Waltman and van Eck, 2015). Still, there seems to be a reluctance to use similar basic rules to evaluate performance of individual researchers.

## H-Index

H-index (Hirsch, 2005) is probably the most popular bibliometric indicator that has been advertised as a measure of individual scientist's output. This index uses a clever combination of publication and citation counts that discounts few accidentally high citation results and makes the indicator more robust compared to simple total citations. However, the promise to quantify individual's performance is misleading. Being based on traditional whole counts, this index cannot adequately handle the problem of multiple authors. Interestingly, Hirsch (2005) admits the problem and the necessity to correct it. Egghe (2008), Schreiber (2008), and Harzing et al. (2014) have introduced the required corrections. Still, the popular providers of citation data present the original (uncorrected) h-index only.

## Highly Cited Researchers

Every year, Clarivate publishes a list of highly cited researchers, the well-known ranking of individual scientists based on traditional whole-count bibliometrics. This list includes the authors of the articles that rank in the top 1% by citations for research field and year of publication. These authors are often linked to notions of scientific excellence or breakthrough research. In a recent study, Aksnes and Aagaard (2021) analyzed publication, citation, and collaboration statistics of these people. The results show that highly cited researchers are usually not those who are credited for individual extraordinary contribution. Rather, they are members of big consortia who publish together with large numbers of coauthors (average number of authors of the highly cited articles was 59, while the average number of authors for all articles in WoS was 4.8). Also, highly cited researchers were remarkably productive—they coauthored, in average, 15 papers per year. Aksnes and Aagaard (2021) also showed that performance measures and chosen top individuals could be fundamentally different when fractionalized publication and citation counts were used instead of whole counts.

Analysts from Clarivate, the publisher of the list of highly cited researchers, have been worried that among highly cited authors, there are too many who mostly publish with hundreds of coauthors (e.g., Clarivate, 2020). Admitting that this observation “strains their reason,” they have tried to reduce the number of these cases. Instead of direct exclusion of papers with too long a list of authors, the papers with more than 30 affiliations of the authors were excluded. This helped to remove a part of “too heavily collaborating” people from the list of highly cited researchers.

This kind of correction looks rather problematic. If the number of coauthored highly cited papers is the indicator of scientific success, then a rational person should maximize both citability and number of coauthored articles. With limited personal resources, the number of articles one can participate in is proportional to the average number of coauthors of these articles. Hence, maximizing collaboration is a necessary means to maximal personal success in terms of Clarivate. Setting limits to this does not solve the real problem.

To break the unintended proportionality of success score with number of coauthors, it is necessary to replace whole counts with fractionalized ones. Fractionalization does not set any limits to the number of authors but removes the motivation to increase the number of collaborators when this is not justified by the nature of research.

Although there are huge differences in numbers of coauthors between the fields of research, the whole-count bias can be expressed by the same simple arithmetic for all of these. Therefore, it is not necessary to invent different measures for scientific disciplines with small and large co-authorship numbers. The simple division is appropriate for all disciplines and supports interdisciplinary comparison as well.

## A Revisionist From Italy

While professional researchers of bibliometrics usually prefer to ignore the problem of multiple authors, there is one remarkable exception.

Giovanni Abramo (Abramo et al., 2013; Abramo and D'Angelo, 2014, 2016) is one of the few professionals in bibliometrics who believe that dividing articles and citations between multiple authors should be obligatory and has used that in practical evaluation of researchers and institutions in Italy. He also tries to take into account different contributions of coauthors, when possible. His ultimate goal is a microeconomic model that measures cost efficiency of science. Critics have argued that this project requires data that are either unavailable or of insufficient quality (Waltman et al., 2016). While the whole project may be too ambitious and difficult to apply worldwide, some of its important parts could be easily applied everywhere. For example, fractionalized counting of publications does not need the gathering of any new data or difficult analysis. At least, the example of Italy proves that division of publications and citations between co-authors has no catastrophic consequences to scientific work. It is yet to be seen if proper measurement of scientific performance gives any advantages to Italian academia.

## Increasing Number of Coauthors

During the last 50 years, the number of authors of a scientific article has steadily increased in every field of science (Wuchty et al., 2007; Adams et al., 2019). Frequently, increasing numbers of coauthors has been seen as a normal process caused by increasing complexity of scientific research and improvement of the means of communication. Some studies have tried to test possible mechanisms of rising numbers of coauthors. For example, Tilak et al. (2015) tested a hypothesis that a large number of coauthors in medical research is caused by increasing complexity of research design. This study revealed a comparable increase of authorship numbers for different complexity levels and concluded that increasing complexity of research cannot explain increasing co-authorship.

There are people who perceive this process as problematic and potentially detrimental. Obviously, large groups spend more energy for organizing and supervising and may be too restrictive for those who want to pursue their own innovative ideas. Nobel Prize winners Hubel (2009) and Higgs (2013) have critically commented the trends in research culture that might not support a kind of pursuit that made their discoveries possible.

Several analysts relate increasing authorship numbers with tough competition, quantitative methods of evaluation, and gratuitous authorship (Lozano, 2013; Von Bergen and Bressler, 2017). Many scientific journals have introduced measures to discourage publishing articles with large number of coauthors. A usual measure requires describing the contribution of each author. It is unlikely that these methods help to set limits to yet unstoppable growth of numbers of co-authors per article.

I agree that increasing number of researchers, their specialization, and available communication technologies make collaboration much more feasible. Still, I believe that biased bibliometric evaluation plays an important role in global increase of numbers of coauthors, as well.

## Does Collaboration Increase Quality?

Several studies have found that increase in number of coauthors correlates with increase of citations (Wuchty et al., 2007; Adams et al., 2019). This has been interpreted as evidence for a positive effect of collaboration on research quality. However, positive correlation between citation and number of coauthors does not necessarily mean that collaboration improves quality of research. There are many other mechanisms that may explain this correlation— for example:

More promising research ideas may attract more people to participate in the project.

Authors who have proven their ability to write highly cited articles are welcome to many groups of collaboration and may choose bigger ones.

Multi-author articles may be perceived as more trustworthy because they supposedly express consensus of many experts and therefore are cited more frequently.

In a broader view, a moderate increase of “quality” may be accompanied with even larger drop of efficiency (for example, we need 10 times more coauthors to increase citation score by factor of 2 or so, e.g., Adams et al., 2019).

## Toward an Optimal Collaboration

Hardly anybody denies that science is essentially a social phenomenon. Every discovery is based on the work of many other people, contemporary and of earlier generations. There is a folk wisdom that two heads are better than one, and frequently, hard problems can be solved by a collective effort. Therefore, it is very natural to think that collaboration is good for science.

Opponents of fractionalized indicators often argue that division of contributions might reduce motivation to collaborate. I agree, but I don't believe that unlimited growth of collaboration makes science better. Bigger groups require more resources for coordination and tend to become less flexible and more bureaucratic. Also, there are statistical studies showing that groundbreaking discoveries have been more frequently published by relatively small groups of co-authors (Wu et al., 2019; Li et al., 2020).

Perhaps there is optimal size of research groups that depends on scientific field, research problem, and personal characteristics of researchers. We could move toward that optimum if we credit researchers for quality and quantity of their scientific results without any confounding variables. Note that fractionalized counting is in fact neutral regarding group size. Rather opposite to that, the present whole-count system rewards researchers working in larger groups beyond their scientific contribution. Therefore, it excludes converging to the optimum group size.

## Relations to Other Problems

Of course, there are many problems in evaluative bibliometrics. Some of these have been successfully studied, some are too complex to solve, and some need data that are difficult to acquire.

During the last 20 years, researchers of bibliometrics have tried hard to make citation scores of articles in different scientific fields comparable (Schubert and Braun, 1996; Waltman and Van Eck, 2013). For that purpose, several methods of normalization have been developed that are now standard options in citation analysis. However, the main goal of bibliometrics is not to evaluate publications but performance of researchers, research groups, or institutions that have contributed to many different publications. Therefore, normalization of citation scores of articles needs to be complemented with a plausible division of credits between coauthors of the articles.

A frequent argument against division of credits by the number of authors is that this assumes equal contributions of the co-authors. However, note that the present whole-count system makes the same assumption. Division of credit leaves the equality problem as it was, but effectively removes the inflation bias related to number of coauthors. Of course, it could be better to use actual contributions of coauthors, but lack of this information should not preclude the correction of the other problem based on currently available information. Specific models of unequal fractionalization (e.g., arithmetic, harmonic) also require additional information and could be a subject of future studies.

Correction of the multi-authorship bias probably leads to some reappraisal of earlier studies where whole-count indicators have been used as a measure of individual's performance. Hopefully, that results in a more consistent picture of the world of science.

## Indicators as a Part of the Environment

For several decades, publication and citation scores have been a part of the environment for scientists. In majority, they have believed that the mainstream indicators are correct measures of success in science and have made their important decisions based on that. The smartest have found how to use the bias of indicators for their personal success. Still, an average researcher has no doubt that the present whole-counting indicators describe accurately the real world and that the higher scores of individuals working in bigger groups show that cooperation is an efficient way to produce good science.

Besides research methods, young researchers in the beginning of their career learn useful tips of how to find influential collaborators or join prestigious consortia. They also acquire unwritten rules of using “honorary,” “gift,” and other forms of authorship. Due to the whole-counting system, original authors have an unlimited amount of currency in a form of potential co-authorship that can be used to pay for various services, support colleagues, thank supervisors, and advance future collaboration. If at least part of these “payments” are reciprocal, your publication and citation scores will grow, without any real cost.

The biased feedback has a pressure on different aspects of science. It looks normal that a lone thinker cannot be very successful in the modern science, because social skills are important in collaboration games. Also, research problems that require a lot of fieldwork and gathering large datasets are preferred over these that primarily need individual imagination and personal dedication.

Is it possible to change this world? One cannot be very optimistic.

For many scientists, it would be difficult to accept a different view of the world that requires learning new rules and possible reappraisal of earlier achievements. People and organizations who have been well-adapted to the present system tend to be reluctant to revision of the rules. Note that, because of a “natural selection,” majority of influential people in science are those who have been best adapted to the current measures of success. Most likely, they do not support the change.

## Conclusions

Behind many problems of contemporary evaluative bibliometrics is inadequate handling of multiple authorship. To move toward theoretically plausible and practically more useful bibliometrics, we should replace default whole-count indicators with fractionalized ones. It requires making the fractionalized indicators readily available in well-known databases and informing scientific community more clearly about their meaning and purpose. Realization of this idea is difficult, because the traditional indicators have become an integral part of our research culture and many people and organizations see the possible change as detrimental to their interests.

## Data Availability Statement

The original contributions presented in the study are included in the article/supplementary material, further inquiries can be directed to the corresponding author/s.

## Author Contributions

EP conceived the idea and wrote this article.

## Conflict of Interest

The author declares that the research was conducted in the absence of any commercial or financial relationships that could be construed as a potential conflict of interest.

## Publisher's Note

All claims expressed in this article are solely those of the authors and do not necessarily represent those of their affiliated organizations, or those of the publisher, the editors and the reviewers. Any product that may be evaluated in this article, or claim that may be made by its manufacturer, is not guaranteed or endorsed by the publisher.
